# Effects of Intermittent Fasting on Cardiometabolic Health: An Energy Metabolism Perspective

**DOI:** 10.3390/nu14030489

**Published:** 2022-01-23

**Authors:** Manuel Dote-Montero, Guillermo Sanchez-Delgado, Eric Ravussin

**Affiliations:** 1 Pennington Biomedical Research Center, Baton Rouge, LA 70808, USA; manueldote@ugr.es (M.D.-M.); eric.ravussin@pbrc.edu (E.R.); 2 PROmoting FITness and Health through Physical Activity Research Group, Department of Physical Education and Sports, University of Granada, 18071 Granada, Spain

**Keywords:** cardiovascular health, metabolic rate, energy expenditure, fat oxidation, alternate-day fasting, twice-weekly fasting, 5:2 diet, modified periodic fasting, fasting-mimicking diet, time-restricted eating, Ramadan, exercise

## Abstract

This review summarizes the effects of different types of intermittent fasting (IF) on human cardiometabolic health, with a focus on energy metabolism. First, we discuss the coordinated metabolic adaptations (energy expenditure, hormonal changes and macronutrient oxidation) occurring during a 72 h fast. We then discuss studies investigating the effects of IF on cardiometabolic health, energy expenditure and substrate oxidation. Finally, we discuss how IF may be optimized by combining it with exercise. In general, IF regimens improve body composition, ectopic fat, and classic cardiometabolic risk factors, as compared to unrestricted eating, especially in metabolically unhealthy participants. However, it is still unclear whether IF provides additional cardiometabolic benefits as compared to continuous daily caloric restriction (CR). Most studies found no additional benefits, yet some preliminary data suggest that IF regimens may provide cardiometabolic benefits in the absence of weight loss. Finally, although IF and continuous daily CR appear to induce similar changes in energy expenditure, IF regimens may differentially affect substrate oxidation, increasing protein and fat oxidation. Future tightly controlled studies are needed to unravel the underlying mechanisms of IF and its role in cardiometabolic health and energy metabolism.

## 1. Introduction

**Figure 1:**
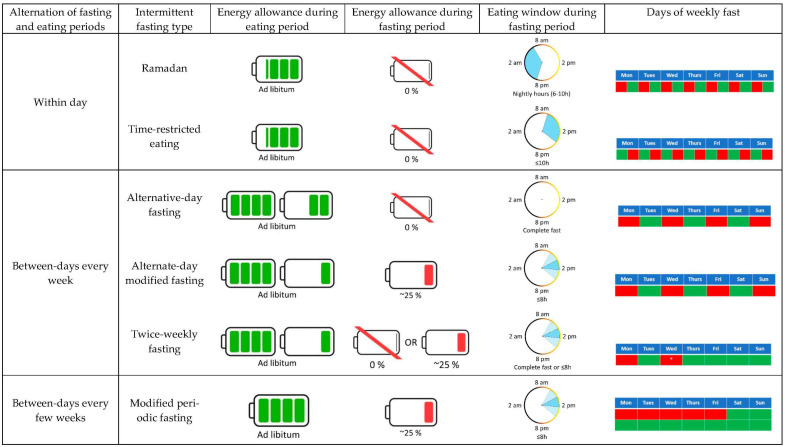
Different types of intermittent fasting. The green and red shading within the battery symbols illustrate the energy intake during the eating and fasting periods, with one battery symbol representing 100% of energy needs. The alternative-day modified fasting, twice-weekly fasting, and modified periodic fasting approaches do not typically restrict food intake to a specific time during the fasting days; therefore, the reduced food intake during these days can be consumed within a single meal (darker blue areas) or various meals (lighter blue areas). The blue areas within the circles indicate the eating window. The areas shaded in red in the calendar tables indicate fasting periods, while the green areas indicate eating periods. * Fast days in the twice-weekly fasting may be consecutive or not.

Caloric restriction (CR), a sustained reduction in energy intake while maintaining optimal nutrition, is probably the most effective non-pharmacological intervention to extend healthspan [1,2]. However, prescribing continuous daily CR results in poor long-term adherence [3], which can be attributed to biological (e.g., increased appetite), behavioral (e.g., social events), psychosocial (e.g., elevated food reward), and environmental (e.g., availability of high-caloric palatable foods) factors [1,2,3]. Over the past decade or so, intermittent fasting (IF) has emerged as a promising alternative to continuous/traditional CR [4]. In simple terms, IF consists of alternating fasting and unrestricted eating periods, which may facilitate adherence [5]. As shown in Figure 1, IF can be categorized into six different approaches: religious fasting, alternative-day fasting (ADF), alternative-day modified fasting (ADMF), twice-weekly fasting usually called the “5:2 diet”, modified periodic fasting usually called “fasting-mimicking diet”, and time-restricted eating (TRE). Religious fasting integrates a myriad of modalities, among which Ramadan is probably the most extended form. While doing Ramadan, Muslims abstain from all forms of food and drink during the daylight hours, thus concentrating all food and liquid intake during the night [6]. ADF consists of alternating 24-h fast periods with the unrestricted intake of food during the subsequent 24 h, repeating that for multiple days or weeks. Similarly, the ADMF modality severely limits food intake (usually to 25% of habitual energy needs or ~500 kcal/day) during 24-h periods, followed by unrestricted access to food for 24 h. Whereas ADF and ADMF usually alternate fasting and fed days in 48-h cycles, the twice-weekly fasting modality limits food intake for two days a week (consecutive or non-consecutive) with complete fasts or with severely restricted energy intake during these days. The modified periodic fasting consists of consuming a plant-based, very-low-caloric diet during 5 consecutive days followed by at least 10 days of unrestricted eating. Lastly, TRE consists of restricting the daily energy intake to a pre-determined eating window (generally ≤10 h), fasting for the rest of the day (14 h or more).

Adherence to IF effectively decreases energy intake, producing weight loss in most studies [7]. Moreover, recent studies suggest that IF improves cardiometabolic health even with no reduction in energy intake [8], challenging the dogma that CR is a prerequisite for IF to induce health benefits. In this review, we will summarize the existing evidence in relation to the effects of different types of IF on human cardiometabolic health. We will emphasize how IF impacts energy metabolism, as this is likely mediating, at least in part, the cardiometabolic health benefits of IF.

## 2. Acute Effect of Fasting on Energy Metabolism

Sustained periods of food scarcity were highly common over the course of human evolution [9,10]. Accordingly, humans have developed numerous behavioral and physiological adaptations that allow them to survive in a food-deprived/fasted state. Contemporary scientific investigation of human starvation began in the late nineteenth and early twentieth centuries [9,11,12,13,14]. We focus our analysis on the coordinated metabolic responses of adults to short-term fasting (i.e., 0–72 h), as it is applicable to all IF regimens.

**Figure 2:**
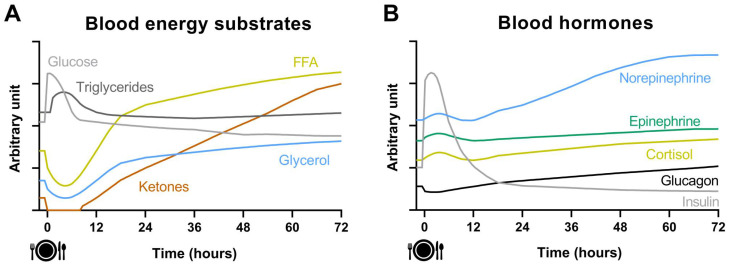
Dynamic changes of blood energy substrates (Panel (**A**)) and blood hormones (Panel (**B**)) during a 72 h fasting period after consuming a meal following an overnight fast.

**Figure 3:**
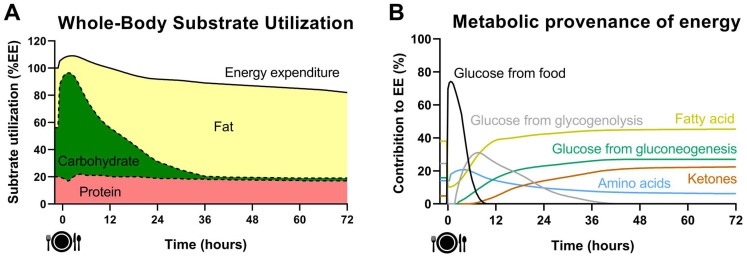
Dynamic changes of whole-body substrate utilization (Panel (**A**)) and metabolic provenance of energy (Panel (**B**)) during a 72-h fasting period after consuming a meal following an overnight fast.

Figure 2 illustrates the dynamic changes of circulating energy substrates and hormones during a 72 h fast, whereas the changes of whole-body substrate utilization and metabolic provenance of energy are illustrated in Figure 3. After a meal consumption, an abrupt increase in blood glucose concentration is detected within ~15 min peaking 30–60 min after the start of the meal [15]. In response to this, beta cells of the pancreatic islets secrete insulin resulting in a drastic increase in systemic insulin (~400–500 pmol/L), while alpha cells decrease the secretion of glucagon. Circulating cortisol and catecholamines concentrations also increase after a meal in response to a macronutrient-dependent stimulation [16,17]. The elevated insulin concentration acts on adipose tissue to inhibit the release of glycerol and free fatty acids (FFA), whose circulating concentration is reduced to ≤0.1 mol/L. This in turn stops the production of ketones that become undetectable in blood [15]. Hepatic glycogen metabolism switches from breakdown (glycogenolysis) to synthesis (glycogenesis) and muscle metabolism from fatty acids and amino acids oxidation to glucose oxidation and glycogen storage. Consequently, carbohydrate utilization represents 70–75% of energy expenditure after consuming a meal. This finely tuned response results in a decrease in blood glucose concentration to < 7.8 mmol/L two hours after a meal [15]. In contrast to the rapid absorption of glucose or amino acids after the meal, the absorption of dietary triglycerides is much slower. The peak in plasma triglycerides concentration (1.5–2 mmol/L) occurs 3–5 h after the meal. Unlike carbohydrate or protein, ingested triglycerides have no or very little influence on their own oxidation and are primarily directed towards deposition in the adipose tissue [18,19]. Food intake induces a 5–15% increase in energy expenditure over a 3–5-h period, known as the thermic effect of food (TEF). TEF is mainly related to the energy cost of digestion, absorption, and storage of the ingested nutrients, although a facultative component of TEF also exists mostly associated with hormonal and autonomic nervous changes [20,21].

Five or six hours after the meal, the postabsorptive state is set, slowly drifting energy metabolism to a state of overnight fasting (~12 h). At that time, blood insulin concentration is low, typically around 60 pmol/L, although it widely varies between individuals [15]. Blood glucose homeostasis (4.0–5.5 mmol/L) is now maintained by counter-regulatory hormones such as glucagon (20–25 pmol/L), cortisol (138–690 nmol/L), epinephrine (<328 pmol/L) and norepinephrine (709–4019 pmol/L), all slightly elevated [15,22]. In this state, carbohydrate oxidation typically represents ~35% of energy expenditure, whereas lipid oxidation is the major contributor to energy expenditure (~45%) [15,23,24]. The remaining ~20% of energy expenditure is covered by protein oxidation, facilitated by proteolysis. Some of the amino acids released (branched-chain amino acids) are oxidized in the muscle and their amino groups are transferred to pyruvate to produce alanine. Both alanine and glycerol are taken up by the liver as substrates for gluconeogenesis. Simultaneously, low insulin levels and circulating catecholamines promote lipolysis with the release of FFA and glycerol from adipose tissue. The concentrations of blood FFA, glycerol and triglycerides are typically ~0.5 mmol/L, ~0.15 mmol/L and ~1 mmol/L after an overnight fast [15]. At this time, ketone bodies, 3-hydroxybutyrate and acetoacetate, are also produced in the liver and secreted into the circulation, although their concentration remains low, usually, ≤0.5 mmol/L for both ketones combined [15].

Beyond the 12 h fasting state, blood glucose and insulin concentration continue to slowly decline, whereas catabolic hormones, FFA, glycerol, and ketones concentrations continue to rise [15,25]. Liver glycogen content decreases drastically within 24 h, and its contribution to energy expenditure becomes almost nil after ~36 h of fasting [26]. As glycogenolysis is reduced, increased gluconeogenesis is triggered to avoid a severe decrease in systemic glucose that would seriously impair brain and other glucose-requiring systems (e.g., erythrocytes) functions [14]. A progressive switch from glycogenolysis to gluconeogenesis is necessary to produce glucose within the first 24 h of fasting [15,26], the time at which gluconeogenesis accounts for ~64% of total glucose production [26]. In parallel, lipolysis is steadily increased, and lipid oxidation becomes the major contributor to energy expenditure. Moreover, the high influx of FFA to the liver in the context of low circulating insulin sharply increases ketogenesis, which is reflected in a continuously increasing circulating concentration of ketones [14,15,25]. Finally, it is worth noting that energy expenditure decreases ~10% after 36–48 h of fasting [27,28] mainly due to the absence of TEF and a low sympathetic tone.

During the first 24–48 h of fasting the main gluconeogenesis precursors are lactate (~50%) and proteolysis (~40%), while the contribution of glycerol is minimal (~10%) [29]. At this pace, with the brain requiring 100–120 g of glucose per day, the body’s stores of protein would be rapidly depleted. Unlike fat stores, body proteins are functionally important, and their progressive depletion leads to major complications and potentially death. Therefore, a series of metabolic adaptations that lead to the sparing of muscle protein kick in from 48–72 h of fasting onwards [14,15,25]. Blood ketones concentration is similar to FFA (~1.5 mmol/L) after 72 h of fasting and the brain begins to use significant amounts of ketones as a fuel source, reducing the need for glucose production [14,15]. In addition, there is a further decrease in metabolic rate beyond the absence of TEF, which is partially underlined by decreased circulating leptin and thyroid hormones [11,15,25].

The metabolic switch from glycogenolysis to gluconeogenesis, fat oxidation and ketogenesis (see Figure 3) seems to be one of the main drivers of the metabolic health benefits provided by IF [30]. TRE extends overnight fasting to 14–20 h, which means that this metabolic switch has just occurred by the end of the fasting period. A slightly more pronounced switch from glycogenolysis to gluconeogenesis occurs with ADMF and twice-weekly fasting since both regimens are characterized by two 14–18 h fasting periods interspersed with short eating windows. ADF is characterized by a further extension of fasting to 24–36 h, implying that liver glycogen content is practically depleted, and the metabolic switch was completely achieved. As discussed in Section 5, exercise may advance the occurrence of the metabolic switch [30,31,32]. Future IF studies should employ new technology such as continuous glucose, FFA and ketone monitoring to better understand the role of the metabolic switch in improvements of metabolic health. As for any other physiological stimuli, the acute metabolic response to fasting is likely to be modified over time after prolonged adaptation to IF and may eventually alter its impact on health and metabolism.

## 3. Impact of IF on Cardiometabolic Health

IF has recently received a lot of attention within the scientific community, as well as among the media and the lay public. Nevertheless, most of the published articles studying the effects of IF on human cardiometabolic health are preliminary studies often with methodological limitations. In this section, we summarize the available evidence for the chronic (≥2 weeks) effects of IF regimens on body weight and composition, ectopic fat, and accepted cardiometabolic risk factors.

### 3.1. Body Weight and Composition

A meta-analysis including 85 studies (4176 adults aged 16–80 years) has shown that Ramadan induces a small reduction in body weight (~−1.0 kg) [33]. This is in line with two other meta-analyses showing that, in the short term (i.e., ≤3 months), IF regimens cause a moderate decrease in body weight of ~−3.0 kg compared to ad libitum eating control groups [34,35]. More specific meta-analyses investigating a particular type of IF have shown that both ADF and TRE triggered body weight loss (~−4.3 kg and ~−0.9 kg, respectively) and fat mass loss (~−4.9 kg and ~−1.6 kg, respectively) compared to ad libitum eating [36,37]. A reduction in fat-free mass was also observed after ADF (~−1.4 kg) [36]. Interestingly, a secondary analysis of a meta-analysis suggested that TRE might be more effective in reducing body weight in metabolically unhealthy participants than in their metabolically healthy counterparts [37].

Whereas both IF and continuous daily CR seem to be effective in reducing body weight and fat mass, how fat-free mass is affected by these two interventions is still a matter of debate [30,38]. According to several meta-analyses, both IF and CR interventions produced similar changes in body weight, fat mass, fat-free mass and waist circumference [7,34,35,39,40,41], provided that the adherence to interventions is similar [7,40,41]. However, a recently published study, imposing a relatively tightly matched degree of energy restriction, has reported that ADF induced lower fat and greater fat-free mass loss (~−0.74 kg and ~−0.75 kg, respectively) than an isocaloric continuous daily CR (~−1.75 kg and ~−0.03 kg, respectively) during 3 weeks in 12 lean healthy adults [42]. Further research with a larger sample size and implementing isocaloric interventions in people with overweight/obesity is needed to unravel the effects of IF regimens on body composition, as compared to continuous daily CR.

### 3.2. Ectopic Fat

Beyond the total amount of body fat, its distribution plays a key role in the pathogenesis of cardiometabolic diseases. Ectopic fat depositions, defined as the accumulation of triglycerides within cells (or sometimes around) of non-adipose tissues (liver, skeletal muscle, beta cells …) are at the center of metabolic health derangements [43]. Only two studies have investigated the effect of IF on ectopic fat assessed by magnetic resonance imaging and/or spectroscopy. Trepanowski et al. [44] observed a ~0.4 kg reduction in visceral fat mass after 6 and 12 months of AMDF, as compared to a non-food restricted control group. However, this change was comparable to a continuous daily CR group. Likewise, Holmer et al. [45] found that 12 weeks of twice-weekly fasting or low-carbohydrate high-fat diet were superior to the standard of care intervention in reducing hepatic steatosis (~−6.1% and ~−7.2%, respectively) in patients with non-alcoholic fatty liver disease (NAFLD), without differences between the twice-weekly fasting and low-carbohydrate high-fat diets. Studies estimating visceral fat mass by dual X-ray absorptiometry have shown conflicting results, with some studies reporting reductions of visceral fat by ADF and 8 h TRE and others showing no differences between ADF, ADMF, 4 h TRE, 6 h TRE, 8 h TRE, and standard nutrition regimen control groups [42,46,47,48]. The cumulative evidence is still very preliminary and future research is warranted to better understand the effects of IF on different ectopic fat depots (e.g., visceral fat, liver fat, intramuscular fat, pancreatic fat) and how it compares to the effects of matched continuous daily CR.

### 3.3. Cardiometabolic Risk Factors

A recent meta-analysis including 91 studies has shown that Ramadan produces a small but significant reduction in serum triglycerides, total cholesterol, low-density lipoprotein cholesterol (LDL-C), diastolic blood pressure, and an increase in high-density lipoprotein cholesterol (HDL-C) [49]. Two other meta-analyses have shown that IF regimens reduced total cholesterol and systolic blood pressure compared to unrestricted eating, although the clinical relevance of the magnitude of these reductions may be debatable [34,40]. Meta-analyses considering only ADF and TRE studies were also conducted. On the one hand, ADF reduced total cholesterol, LDL-C, triglycerides, and blood pressure, but did not modify HDL-C, fasting glucose or insulin sensitivity [36]. On the other hand, TRE decreased systolic blood pressure, triglycerides, and fasting glucose, but not diastolic blood pressure, LDL-C and HDL-C, which might be dependent on the baseline metabolic health status of study participants [37,50]. When compared to continuous daily CR, IF regimens were equally effective in improving plasma glucose, glycated hemoglobin, triglycerides, total cholesterol, LDL-C, HDL-C, systolic and diastolic blood pressure, or C-reactive protein [34,40]. Nevertheless, one study conducted in our lab found that eucaloric (i.e., energy intake equals energy needs, and thus in absence of weight loss) early TRE impressively improved blood pressure while still improving oxidative stress, insulin sensitivity, and β cell responsiveness in men with prediabetes [8]. Overall, different modalities of IF were shown to improve the cardiometabolic risk profiles of human adults. Future larger studies will probably elucidate whether IF has weight-loss independent effects on cardiometabolic risk factors, how it compares to continuous daily CR, and whether different IF modalities exert different beneficial effects.

The majority of IF studies have measured only fasting biomarkers of cardiometabolic risk. However, assessing energy metabolism during the post-prandial state is key for understanding the effect of an intervention on the overall cardiometabolic risk as humans in modern societies spend most of the day in a postprandial state [51]. Indeed, postprandial glycemia, insulinemia and lipidemia are all implicated in the etiology of chronic cardiometabolic diseases [52,53]. Importantly, Antoni et al. [54] showed that twice-weekly fasting was superior to continuous daily CR in reducing postprandial triglycerides following matched weight loss of 5%. Similarly, Templeman et al. [42] have recently reported that 3 weeks of ADF producing a ~25% energy deficit reduced postprandial triglycerides in lean healthy adults, whereas an increase was observed by continuous daily CR producing the same energy deficit. However, no significant differences were detected in postprandial glucose, insulin, FFA and glycerol in both studies [42,54]. Similar results were observed between 2-week interventions of either a twice-weekly fasting or continuous daily CR in normal-weight, young adults [55]. Lastly, a 4-day crossover study carried out in our lab found that eucaloric (i.e., energy intake equals energy needs, and thus in absence of weight loss) 6 h early TRE decreased mean 24 h and nocturnal glucose levels [56]. In the same vein, Parr et al. [57] conducted a 5-days crossover study in isocaloric conditions, showing that early TRE reduced nocturnal glucose concentrations. In contrast, another 7-day crossover study found no effect of early TRE and late TRE in the mean 24 h glucose levels [58]. Similarly, 10 h and 8 h self-selected TRE did not improve mean glucose levels [59,60] after 12 weeks of intervention. Therefore, it appears that only early TRE improves glycemic control, although further studies assessing postprandial metabolism and including continuous glucose monitoring are needed to confirm this hypothesis.

### 3.4. Impact of IF on Cardiometabolic Health: Conclusion and Gaps

Overall, IF regimens seem to improve body composition, ectopic fat, and classic cardiometabolic risk factors compared to ad libitum eating control groups. However, IF does not seem to provide additional benefits when compared to continuous daily CR, suggesting that if a net energy deficit is achieved, it becomes the main driver of the cardiometabolic health benefits. Therefore, energy balance needs to be matched and tightly controlled (e.g., food being provided and/or energy intake being objectively measured by methods such as the intake-balance method using doubly-labeled water) to allow a proper comparison of both interventions and really isolate the effects of fasting and energy restriction on cardiometabolic health, which was performed only in a few studies [8,42]. However, preliminary studies suggest that IF might be beneficial in the absence of weight loss [8]. Consequently, IF may be useful to enhance cardiometabolic health of weight stable individuals, for instance, after a weight loss intervention.

The underlying mechanisms of fasting are thought to be mediated, at least in part, by the metabolic switch from carbohydrate utilization to fat and ketones oxidation that happens during fasting (see Figure 3) [30]. The capacity to rapidly adjust substrate oxidation to substrate availability and energy demand, known as metabolic flexibility, is considered central in the prevention of ectopic fat accumulation, which in turn induces peripheral insulin resistance [61,62]. Therefore, future studies need to be designed to test whether IF regimens improve metabolic flexibility leading to a greater loss (or less accumulation) of ectopic fat depots, thus enhancing biomarkers of cardiometabolic risk. However, a potential side effect of the metabolic switch associated with IF regimens may be elevated proteolysis to supply gluconeogenesis during the fasting periods, causing excessive fat-free mass loss which is known to impair physical function and cardiometabolic health and favor weight regain and increased fatness [42,63,64]. It is therefore important to determine whether fat-free mass loss differs between IF regimens and continuous daily CR. Lastly, future IF studies should employ wearable technologies such as continuous glucose, FFA and ketone monitoring, accelerometers to assess physical activity and sleep, skin temperature sensors, and smartphone apps to assess dietary intake. Such data will provide a better understanding of the effects of IF on cardiometabolic health.

## 4. Effects of IF on Energy Metabolism: Energy Expenditure and Substrate Oxidation

Energy metabolism (i.e., energy expenditure and the substrates oxidized to sustain energy expenditure) is crucially linked to cardiometabolic health. For instance, a low energy expenditure, adjusted for body size and composition, and a reduced-fat oxidation rate predict future weight gain [65,66,67]. Likewise, changes in energy expenditure and substrate oxidation in response to perturbations of energy intake were shown to be determinants of weight gain, and weight loss during controlled dietary interventions or in free-living conditions [68,69]. As discussed in Section 2, fasting acutely impacts energy metabolism. Therefore, it is plausible that adaptations in energy metabolism are part of the underlying mechanisms by which IF exerts benefits on cardiometabolic health. In this section, we summarize the existing evidence on the effects of different modalities of IF on energy expenditure and substrate oxidation.

### 4.1. Ramadan

Despite the numerous studies conducted during Ramadan, only three have documented its impact on energy expenditure and/or substrate oxidation (Table 1) [70,71,72,73]. These studies have reported no changes in resting metabolic rate (RMR) after Ramadan [71,72,73]. Nonetheless, Lessan et al. [72] found that RMR, but not total daily energy expenditure (TDEE) assessed by doubly-labeled water, was significantly decreased during the 3 last weeks of the 4-week Ramadan period. Regarding substrate oxidation, one study observed similar fasting respiratory exchange ratio (RER) values before and after Ramadan [73], whereas Lessan et al. [72] detected a significant reduction in fasting RER (from 0.88 to 0.80) after Ramadan.

### 4.2. Alternate-Day Fasting (ADF)

A total of seven studies have assessed the effects of ADF on energy expenditure and/or substrate oxidation (Table 2) [42,74,75,76,77,78]. Studies analyzing the effects of ADF on RMR have yielded inconsistent results. In general, short ADF interventions (≤8 weeks) do not seem to affect RMR [42,74,76,78]. Only one 2-week ADF intervention study has shown a reduction in RMR even in the absence of body mass loss [77]. Two studies have compared the changes in RMR induced by ADF and continuous daily CR. Templeman et al. [42] found no differences between both interventions, whereas Catenacci et al. [74] detected that RMR (adjusted for changes in fat-free mass and fat mass) decreased after 8-week of continuous daily CR (~−111 kcal/day), but not after ADF (~−16 kcal/day). On the other hand, fasting substrate oxidation seems not to be affected by short-term ADF interventions [42,75,77]. However, Heilbronn et al. [76] observed that substrate oxidation during the fed days was comparable to baseline values, but fasting carbohydrate oxidation was decreased and fat oxidation increased during the fasting days. Lastly, Templeman et al. [42] observed greater postprandial fat oxidation in two ADF groups (with and without CR) compared to continuous daily CR after 3 weeks of intervention. Clearly, the currently available evidence is limited and further, properly powered, long-term, controlled clinical research is needed to really understand the effects of ADF on energy expenditure and substrate oxidation.

### 4.3. Alternate-Day Modified Fasting (ADMF)

We identified three studies in which energy expenditure and/or substrate oxidation were measured in response to ADMF (Table 2) [79,80,81]. All of them found no significant impact of ADMF on RMR or adjusted 24 h energy expenditure, adjusted activity energy expenditure, and adjusted sleeping energy expenditure between ADMF and continuous daily CR. Moreover, Coutinho et al. [80] observed similar fasting substrate oxidation between ADMF and continuous daily CR after 12 weeks of intervention.

### 4.4. Twice-Weekly Fasting

Three studies comparing the effects of twice-weekly fasting versus continuous daily CR reported no significant differences on RMR and/or RER in adults with overweight or obesity [54,82] or in healthy individuals with normal weight (Table 2) [55]. The concordance between the results of studies investigating ADF and the twice-weekly fasting can be partly attributed to the similarity in study designs as well as the matching of energy deficit and weight loss in both ADMF and twice-weekly fasting interventions. No studies have compared the effects of ADMF or twice-weekly fasting versus ad libitum eating on energy expenditure and/or substrate oxidation.

**Table 2:** Acute and chronic effects of alternative-day fasting, alternative-day modified fasting, and twice-weekly fasting on energy expenditure and substrate oxidation.

Study	Population	Design	Fasting Regimen	Eating Regimen	Assessment	No Change	Increase	Decrease
Acute alternative-day fasting								
[27]	Healthy normal weight/obesity *n* = 14 (14 M/0 F)	48 h Cross-over (a) Control (b) ADF	(2) 48 h fasting	(1) 100% Energy needs	Metabolic chamber	AEE		24 h EE and SEE 24 h RER
[83]	Healthy normal weight/obesity *n* = 64 (51 M/13 F)	36 h Cross-over (a) Control (b) ADF	(2) 36 h fasting	(1) 100% Energy needs	Metabolic chamber	SEE	24 h Fat ox.	24 h EE 24 h RER and sleep RER 24 h CHO ox. 24 h Protein ox.
Chronic alternative-day fasting								
[75]	Healthy normal/overweight *n* = 8 (8 M/0 F)	2-week Single-arm (1) ADF	20 h fasting every other day (From 10 p.m. to 6 p.m.)	Ad libitum	Overnight fasting Metabolic cart (Oxycon Pro, Jaeger)	RER		
[76]	Healthy normal/overweight *n* = 16 (8 M/8 F)	22-day Single-arm (1) ADF	24 h fasting every other day	Ad libitum	Overnight fasting Metabolic cart (DeltaTrac, SensorMedics)	RMR and adjusted RMR * in fed and fast days RER, CHO and Fat ox. In fed day	Fat ox. In fast day	RER and CHO ox. In fast day
[77]	Healthy normal/overweight *n* = 8 (8 M/0 F)	2-week Cross-over (a) Control (b) ADF	(2) 20 h fasting every other day (From 10 p.m. to 6 p.m.)	1 and (2) 100% Energy needs	Overnight fasting Metabolic cart	RER, CHO and Fat ox.		RMR
[74]	Obesity (1) *n* = 12 (3 M/9 F) (2) *n* = 13 (3 M/10 F)	8-week RCT (1) CR (2) ADF	(2) 24 h fasting every other day	(1) Prescribed CR −400 kcal/day. Measure 28% CR (2) 100% Energy needs + ad libitum access to 5–7 snacks (200 kcal/serve) during eating days. Measure 47% CR	Overnight fasting Metabolic cart (TrueOne 2400, Parvo Medics)	RMR and adjusted RMR *		
[78]	Healthy normal/overweight (1) *n* = 29 (12 M/17 F) (2) *n* = 28 (11 M/17 F)	4-week RCT (1) Control (2) ADF	(2) 24 h fasting every other day	(1) Ad libitum. Measured 8% CR (2) Ad libitum. Measured 37% CR	Overnight fasting Metabolic cart (MetaMax 3b, Cortex)	RMR		
[78]	Healthy normal/overweight (1) *n* = 60 (24 M/36 F) (2) *n* = 30 (14 M/16 F)	Observational (1) Control (2) ADF performed >24 weeks	(2) 24 h fasting every other day	(1) Ad libitum. Measured 0% CR (2) Ad libitum. Measured 29% CR	Overnight fasting Metabolic cart (MetaMax 3b, Cortex)	RMR		
[42]	Healthy normal/overweight (1) *n* = 12 (5 M/7 F) (2) *n* = 12 (3 M/9 F) (3) *n* = 12 (7 M/5 F)	3-week RCT (1) CR (2) ADF without CR (3) ADF + CR	2 and (3) 24 h fasting every other day	(1) Daily 25% CR (2) 200% Energy needs in fed days, 0% net CR (3) 150% Energy needs in fed days, 25% net CR	Overnight fasting and 3-h postprandial period Metabolic cart	RMR and adjusted RMR ^¥^ Fasting CHO, Fat and Protein ox. Postprandial CHO and Protein ox.	Postprandial Fat ox. in both ADF groups vs. CR	
Chronic alternative-day modified fasting								
[81]	Overweight/obesity (1) *n* = 10 (0 M/10 F) (2) *n* = 10 (0 M/10 F) (3) *n* = 10 (0 M/10 F)	4-week Cross-over (a) CR (b) Bread ADMF (c) ADMF	(2) Ad libitum bread, coffee and tea (3) 50% CR	(1) Daily 50% CR (2) 100% Energy needs (3) 100% Energy needs	Metabolic chamber	Adjusted 24 h EE ^¥^ and AEE Adjusted SEE ^¥^ in CR vs. ADMF		Adjusted SEE ^¥^ in ADMF vs. bread ADMF
[80]	Obesity (1) *n* = 14 (4 M/10 F) (2) *n* = 14 (2 M/12 F)	12-week RCT (1) CR (2) ADMF	(2) EI of 660 and 550 kcal/day for men and women, respectively, on 3 non-consecutive days/week	(1) Daily 25% CR (2) 100% Energy needs	Overnight fasting Metabolic cart (Vmax Encore 29N, Care Fusion)	RMR and adjusted RMR ^α^ RER		
[79]	Overweight/obesity (1) *n* = 18 (0 M/18 F) (2) *n* = 12 (0 M/12 F)	RCT ≥ 5% WL within 12 weeks (1) CR (2) ADMF	(2) 75% CR every other day	(1) Daily 25% CR (2) Ad libitum	Overnight fasting Metabolic cart (GEMNutrition)	RMR		
Chronic twice-weekly fasting (2-WF)								
[54]	Overweight/obesity (1) *n* = 12 (6 M/6 F) (2) *n* = 15 (7 M/8 F)	RCT 5% WL target (1) CR (2) 2-WF	(2) 75% CR on two consecutive days/week	(1) Daily 33% CR (2) Ad libitum	Overnight fasting Metabolic cart ^Φ^ (ISGEM319, GEMNutrition)	RMR and adjusted RMR ^£^ RER		
[82]	Adults with central obesity (1) *n* = 22 (6 M/16 F) (2) *n* = 21 (6 M/15 F)	4-week RCT (1) CR (2) 2-WF	(2) EI of 600 kcal on two consecutive days/week	(1) Daily 500 kcal CR (2) Energy-controlled diet to target the same weekly energy deficit as the CR	Overnight fasting Metabolic cart (FitMate, Cosmed)	RMR		
[55]	Healthy normal weight (1) *n* = 8 (4 M/4 F) (2) *n* = 8 (4 M/4 F)	2-week RCT (1) CR (2) 2-WF	(2) 70% CR on two non-consecutive days/week	(1) Daily 20% CR (2) 100% Energy needs	Overnight fasting Metabolic cart (Quark CPFT, Cosmed)	RMR RER		

No change indicates no significant difference to pre-intervention or control group values; increase indicates significantly higher than pre-intervention or control group values; and decrease indicates significantly lower than pre-intervention or control group values. * RMR results adjusted for fat-free mass and fat mass; ^¥^ RMR, 24 h EE or SEE results adjusted for body weight or fat-free mass; ^α^ RMR results adjusted for fat-free mass; ^£^ RMR results adjusted for metabolically active mass (fat-free mass + 18 kg); ^Φ^ Sample size *n* = 10 and *n* = 13 for CR and twice-weekly fasting (2-WF), respectively. Metabolic cart brands and models are provided when they are available. Abbreviations: ADF, alternate-day fasting; ADMF, alternative-day fasting modified fasting; AEE, activity energy expenditure; CR daily calorie restriction; EE, energy expenditure; RER, respiratory exchange ratio; RMR, resting energy expenditure; SEE, sleeping energy expenditure; WL, weight loss.

### 4.5. Time-Restricted Eating (TRE)

TRE is the most studied IF modality when it comes to energy metabolism. This is due not only to the increased popularity of TRE over the past few years, probably fueled by the potential to be widely applicable in clinical settings but also to older studies investigating topics such as breakfast skipping and meal frequency, extending nocturnal fasting and confining all daily eating events to a 4–10 h window. A detailed description of the studies investigating the acute (i.e., ≤72 h) and chronic effects of TRE on energy expenditure and substrate oxidation is provided in Table 3.

We identified eight studies in which energy expenditure was measured in response to acute TRE [84,85,86,87,88,89,90,91]. TRE can be categorized based on when the eating window occurs, that is early TRE (eating earlier in the day), midday TRE (eating in the middle of the day) and late TRE (eating later in the day). It is hypothesized that early TRE may be the most effective schedule to enhance cardiometabolic health since it aligns with metabolism circadian rhythms [8,92]. However, mixed findings between early, midday and late TRE were observed when it comes to energy expenditure. Overall, TRE, independently of the time of the eating window, does not seem to acutely affect energy expenditure [84,85,86,87,88,91,93]. The study of Nas et al. [90] was the only one that reported that both 6 h early and 6 h late TRE led to a small but significant increase 24 h energy expenditure (91 kcal/day and 41 kcal/day, respectively) as compared to an isocaloric 12 h control schedule. Three other studies have found an increase in RMR [85], sleeping metabolic rate [84] or night energy expenditure [91], but not in 24 h energy expenditure as compared to isocaloric control conditions.

A total of seven studies reported substrate oxidation in response to acute TRE [84,85,86,87,88,89,90], providing mixed findings. In general, TRE, regardless of the time of the eating window, does not appear to acutely affect 24 h substrate oxidation [84,85,86,87,88,93]. However, Nas et al. [90] have shown that 6 h late TRE decreased 24 h carbohydrate oxidation and increased 24 h fat oxidation in comparison to 12 h control. No differences were observed between 6 h early TRE and 12 h control, which might contrast with the hypothesis that early TRE is the most effective schedule [8,92]. Nonetheless, it is important to highlight that the duration of the intervention was only 24 h and that there were no significant differences between 6 h early and 6 h late TRE. Lastly, Munsters and Saris [85] noted an increase in protein oxidation in response to 9 h early TRE versus a 13 h eating window (14 meals per day) without differences in carbohydrate or fat oxidation.

Although the acute response to TRE provides valuable knowledge, longer studies are needed to really understand the influence of TRE on energy expenditure and substrate oxidation. We identified 10 studies that have measured energy expenditure in response to TRE interventions ranging from 4 days to 13 weeks [48,94,95,96,97,98,99,100,101,102]. Overall, the length of the eating window and the circadian timing of food intake did not impact 24 h energy expenditure or RMR, which is consistent with findings from acute TRE studies. Only a few studies have assessed the substrate oxidation response to chronic TRE. Ogata et al. [101] found no significant differences in 24 h substrate oxidation between 5:30 h late TRE and 11 h control. Similarly, fasting RER was not altered by a 12-week 8 h late TRE compared to three ad libitum meals per day (~16 h of eating window) [48]. However, we observed an increased 24 h protein oxidation and decreased 24 h nonprotein RER, especially at nighttime, during a 6 h early TRE intervention, which is indicative of elevated fat oxidation [102]. Therefore, it appears that the circadian timing of the eating window may affect substrate oxidation, although long-term and well-powered trials are needed to test this hypothesis.

**Table 3:** Acute and chronic effects of time-restricted eating on energy expenditure and substrate oxidation.

Study	Population	Design and Intervention	Assessment	No Change	Increase	Decrease
Acute time-restricted eating						
[88]	Healthy normal/overweight *n* = 13 (2 M/11 F)	48 h Cross-over (a) 13 h Control (7:30 a.m.–8:30 p.m., 7 meals/day, 100% energy needs) (b) 6 h TRE (12 p.m.–6 p.m., 2 meals/day, 100% energy needs)	Metabolic chamber	24 h EE 24 h RER Fat ox. from 6 p.m.–9 p.m.	Fat ox. from 9 a.m.–12 p.m. CHO ox. from 6 p.m.–9 p.m.	CHO ox. from 9 a.m.–12 p.m.
[91]	Overweight/Obesity *n* = 10 (0 M/10 F)	48 h Cross-over (a) 10 h Control (9 a.m.–7 p.m., 6 meals/day,1000 kcal/day) (b) 8 h TRE (11 a.m.–7 p.m., 2 meals/day, 1000 kcal/day)	Metabolic chamber	24 h EE	Night EE	
[87]	Healthy normal weight *n* = 14 (0 M/14 F)	36 h Cross-over (a) 8:30 h TRE (8 a.m.–4:30 p.m., 3 meals/day, 100% energy needs) (b) 8:30 h TRE (8 a.m.–4:30 p.m., 2 meals/day, 100% energy needs)	Metabolic chamber	24 h EE, SMR, TEF and AEE 24 h Fat, 24 h CHO and 24 h protein balance	24 h Fat ox. in 3 meals/day vs. 2 meals/day	24 h and night RER in 3 meals/day vs. 2 meals/day 24 h CHO ox. in 3 meals/day vs. 2 meals/day
[85]	Healthy normal weight *n* = 12 (12 M/0 F)	36 h Cross-over (a) 13 h Control (8 a.m.–9 p.m., 14 meals/day, 100% energy needs) (b) 9 h TRE (8 a.m.–5 p.m., 3 meals/day, 100% energy needs)	Metabolic chamber	24 h EE, SMR, TEF and AEE 24 h RER, 24 h CHO and 24 h Fat ox.	RMR 24 h Protein ox.	
[84]	Healthy normal/overweight *n* = 8 (8 M/0 F)	24 h Cross-over (a) 11 h Control (8 a.m.–7 p.m., 3 meals/day, 100% energy needs) (b) 7 h TRE (12 p.m.–7 p.m., 2 meals/day, 100% energy needs)	Metabolic chamber	24 h EE, RMR and TEF 24 h RER, 24 h CHO, 24 h Fat and 24 h Protein ox.	SMR Morning Fat ox. Sleeping CHO ox.	Morning CHO ox. Sleeping Fat ox.
[86]	Healthy normal/overweight *n* = 15 (7 M/8 F)	24 h Cross-over (a) 12:30 h Control (9 a.m.–9:30 p.m., 6 meals/day, 100% energy needs) (b) 10 h TRE (9 a.m.–7 p.m., 3 meals/day, 100% energy needs)	Metabolic chamber	24 h EE 24 h RER and 24 h Fat ox.		
[90]	Healthy normal/overweight *n* = 17 (8 M/9 F)	24 h Cross-over (a) 12 h control (7 a.m.–7 p.m., 3 meals/day, 100% energy needs) (b) 6 h TRE (7 a.m.–1 p.m., 2 meals/day, 100% energy needs) (c) 6 h TRE (1 p.m.–7 p.m., 2 meals/day, 100% energy needs)	Metabolic chamber ^Φ^	24 h Protein ox. and balance in both 6 h TRE No significant differences between both 6 h TRE	24 h EE in both 6 h TRE 24 h Fat ox. and 24 h CHO balance in 1 p.m.–7 p.m. TRE	24 h RER, 24 h CHO ox. and Fat balance in 1 p.m.–7 p.m. TRE
[89]	Healthy normal weight *n* = 12 (2 M/10 F)	72 h Cross-over (a) 13 h Control (8 a.m.–9 p.m., 4 meals/day, 100% energy needs) (b) 10 h TRE (8 a.m.–6 p.m., 4 meals/day, 100% energy needs)	Overnight fasting and 4-h postprandial period Douglas bags	RMR and RER		RER 30 and 60 min after a meal
Chronic time-restricted eating						
[94]	Healthy normal/overweight *n* = 8 (8 M/0 F)	2-week Cross-over (a) 10 h Control (9 a.m.–7 p.m., 6 meals/day, 100% energy needs) (b) 8 h TRE (11 a.m.–7 p.m., 2 meals/day, 100% energy needs)	Metabolic chamber	24 h EE and RMR	Night EE	Day EE
[96]	Healthy normal/overweight *n* = 10 (10 M/0 F)	7-day Cross-over (a) 13 h Control (7:30 a.m.–8:30 p.m., 7 meals/day, 100% energy needs) (b) 6 h TRE (12 p.m.–6 p.m., 2 meals/day, 100% energy needs)	Metabolic chamber Doubly-labeled water during 7 days	24 h EE, TDEE, RMR, TEF and AEE		
[95]	Overweight/Obesity *n* = 14 (0 M/14 F)	4-week RCT (1) 13:30 h CR (7:30 a.m.–9 p.m., 3–5 meals/day, 1000 kcal/day) (2) 6 h TRE (12 p.m.–6 p.m., 2 meals/day, 1000 kcal/day)	Metabolic chamber	24 h EE, SEE and TEF		
[97]	Healthy normal/overweight (1) *n* = 16 (6 M/10 F) (2) *n* = 17 (6 M/11 F)	6-week RCT (1) Control (Ad libitum energy intake starting within 2 h of waking) (2) TRE (Ad libitum energy intake starting after 12 p.m.)	Overnight fasting and 2-h postprandial period Metabolic cart	RMR		TEF
[98]	T2DM *n* = 54 (29 M/25 F)	12-week Cross-over (a) CR (6 meals/day, measured −380 kcal/day) (b) 10 h TRE + CR (6 a.m.–4 p.m., measured −420 kcal/day)	Overnight fasting Metabolic cart ^α^ (VMAX, SensorMedics)	RMR		
[99]	(1) Normal weight *n* = 9 (4 M/5 F) (2) Overweight/obesity *n* = 10 (3 M/7 F) (3) Normal weight *n* = 9 (5 M/4 F) (4) Overweight/obesity *n* = 9 (4 M/5 F)	7-day Cross-over (a) Control (Ad libitum energy intake starting within 2 h of waking) (b) TRE (Ad libitum with breakfast skipping)	Overnight fasting and 2-h postprandial period Douglas bag	RMR and 2 h TEF		
[100]	Obesity (1) *n* = 16 (6 M/10 F) (2) *n* = 17 (6 M/11 F)	6-week RCT (1) Control (Ad libitum energy intake starting within 2 h of waking) (2) TRE (Ad libitum energy intake starting after 12 p.m.)	Overnight fasting and 2-h postprandial period Metabolic cart	RMR and TEF		
[101]	Healthy normal/overweight *n* = 9 (9 M/0 F)	6-day Cross-over (a) 11 h Control (7 a.m.–6 p.m., 3 meals/day, 100% energy needs) (b) 5:30 h TRE (12:30 p.m.–6 p.m., 2 meals/day, 100% energy needs)	Metabolic chamber	24 h EE and 7 h SEE 24 h RER, 24 h CHO, 24 h Fat and 24 h Protein ox.		TEF
[102]	Overweight/Obesity *n* = 10 (6 M/4 F)	4-day Cross-over (a) 12 h control (8 a.m.–8 p.m., 3 meals/day, 100% energy needs) (b) 6 h TRE (8 a.m.–2 p.m., 3 meals/day, 100% energy needs)	Metabolic chamber	24 h EE and RMR Day RER	Day EE and TEF 24 h Protein ox.	Nigh EE and sleep EE 24 h RER, night, rest and sleep RER
[48]	Overweight/Obesity (1) *n* = 25 (15 M/10 F) (2) *n* = 25 (13 M/12 F)	13-week RCT (1) ~16 h Control (6–10 a.m. to 5–10 p.m., 3 meals/day, ad libitum) (2) 8 h TRE (12 p.m.–8 p.m., ad libitum)	Overnight fasting Metabolic cart (TrueOne 2400, Parvo Medics) Doubly-labeled water during 7 days	RMR, RER and TDEE		

No change indicates no significant difference to pre-intervention or control group values; increase indicates significantly higher than pre-intervention or control group values; and decrease indicates significantly lower than pre-intervention or control group values. ^Φ^ Sample size *n* = 15; ^α^ Sample size *n* = 52. Metabolic cart brands and models are provided when they are available. Abbreviations: AEE, activity energy expenditure; CR daily calorie restriction; EE, energy expenditure; RER, respiratory exchange ratio; RMR, resting energy expenditure; SEE, sleeping energy expenditure; TEF, thermic effect of food; TRE, time-restricted eating.

## 5. Optimizing IF by Combining It with Exercise

### 5.1. Metabolic Switching: The Role of Endurance Exercise

As previously mentioned, the metabolic switches from glycogenolysis to gluconeogenesis and from carbohydrate to fat oxidation and ketogenesis (see Figure 3) are thought to be key factors promoting some of the health benefits of IF [30]. Importantly, this metabolic switch largely depends on the state of hepatic glycogen content and, to a lesser extent, on muscle glycogen content. In sedentary conditions, the liver glycogen content severely decreases within 24 h of fasting and it is almost depleted after 36–48 h [15,26]. However, liver glycogen content is substantially depleted within 90–120 min of moderate- to high-intensity exercise if it is not compensated by ingesting carbohydrates [31]. Therefore, exercise appears to be an effective strategy to advance, and perhaps potentiate, the metabolic switch from glycogenolysis to gluconeogenesis, fat oxidation and ketogenesis [30,31,32].

To the best of our knowledge, there are no studies explicitly investigating the combined effects of IF and exercise on energy expenditure and substrate oxidation assessed by whole-room indirect calorimetry. However, several studies have been conducted to investigate the effects of exercise on 24 h energy metabolism under energy balance conditions. Some of these studies concluded that a short session (≤1 h) of moderate- or vigorous-intensity exercise performed in the postprandial state does not influence 24 h fat oxidation [103]. In contrast, Schrauwen et al. [104] reported that high-intensity interval exercise until exhaustion (i.e., glycogen-depleting exercise) performed 2 h after eating increased fat oxidation in the subsequent 24 h. Therefore, the intensity of exercise and the resulting glycogen depletion seem to be key factors in modulating 24 h fat oxidation. On the other hand, 24 h fat oxidation was consistently shown to be increased when moderate-intensity exercise (60–100 min) is performed before breakfast, i.e., in a fasted state [105,106,107]. Although the circadian regulation of energy metabolism cannot be disregarded, the combined effect of an overnight fast and exercise on glycogen depletion and the release of FFAs seem to be the underlying mechanism [105,106,107]. Therefore, glycogen-depleting exercise performed during the fasting periods of IF regimes may be the most efficacious combination to boost the metabolic switch from glycogenolysis to gluconeogenesis, fat oxidation and ketogenesis [30,31,32,104,105].

A potential side effect of ADF and TRE is a behavioral compensatory decrease in low- to moderate-intensity physical activity [42,97,100]. Indeed, the energy expended via physical activity is the most fluctuating component of energy expenditure and hence has the greatest potential to undermine an imposed energy deficit. Consequently, performing structured endurance exercise and/or physical activity may be an effective strategy to avoid an IF-induced reduction in total daily energy expenditure.

### 5.2. Optimizing IF by Combining It with Resistance Exercise

Based on the existence of non-caloric deprived periods, some have hypothesized that IF regimens may decrease fat mass while retaining larger amounts of fat-free mass, as compared with continuous CR [30,38]. In contrast, some studies have reported greater fat-free mass loss with IF regimens than with continuous daily CR [42,63,108], which can be attributed to increased proteolysis to supply substrates for gluconeogenesis during fasting periods (see Figure 3). Fat-free mass loss is known to impair physical functionality, cardiometabolic health and may be a risk factor for weight regain and increased fatness [42,63,64]. This side effect of IF may be counteracted, at least in part, by exercise, in particular, resistance training since it is known to increase skeletal muscle mass [109,110].

Indeed, a recent systematic review and meta-analysis including eight studies and 221 participants, have shown that IF regimens combined with resistance exercise reduced fat mass (~−1.3 kg) with preservation of fat-free mass compared to non-CR control diets [111]. Therefore, more longer-term, and high-quality studies are needed to examine whether resistance exercise can mitigate the fat-free mass loss associated with each type of IF. Moreover, these effects may be dependent on the time at which exercise is performed (i.e., fasting versus eating periods), hence it is important to further clarify the most effective and feasible exercise timing in the context of IF.

## 6. Perspectives of the Usefulness of IF on Cardiometabolic Health: Gaps and Future Directions

Energy expenditure is decreased in parallel to weight loss, which is in part, but not completely due to the loss of metabolically active tissues. A further reduction in energy expenditure beyond what can be predicted by changes in body weight and composition (i.e., metabolic adaptation or adaptive thermogenesis) has been consistently documented [112]. Metabolic adaptation is believed to progressively counteract further weight loss and contribute to weight regain [112]. Therefore, preventing metabolic adaptation will likely contribute to a more pronounced and sustainable weight loss. Whether reducing body weight by IF instead of continuous CR prevents metabolic adaptation is still unknown. Some preliminary studies suggest that there are no differences in the change in RMR between the two intervention modes. Nonetheless, not all studies have reported these findings [74,77], and well-controlled studies are needed to determine whether the decreases in the different components of energy expenditure are comparable between IF regimens and continuous daily CR.

Although TRE does not seem to impact 24 h energy expenditure when compared to larger eating windows (≥12 h) [113], the few studies performed using room indirect calorimetry suggest that IF regimens may affect substrate oxidation, increasing protein and fat oxidation. In normal conditions, carbohydrate and protein oxidation are closely regulated to match their intake, whereas the gap between energy intake and expenditure is buffered by fat balance [114,115]. Furthermore, a reduced-fat oxidation rate in energy balance conditions or in response to acute overfeeding is predictive of long-term weight gain and is thought to determine ectopic fat accumulation [61,66,68]. Therefore, increasing fat oxidation by TRE with or without exercise might prevent weight gain and ectopic fat deposition. Long-term and well-powered trials are needed to ascertain whether IF regimens increase fat oxidation and if this ultimately leads to better weight management and metabolic health than continuous daily CR.

The cardiometabolic health benefits of IF regimens may be optimized, and its undesirable effects attenuated, by combining it with exercise [111]. For instance, adding exercise training to an IF approach may advance by several hours, and probably reinforce, the metabolic switch from glycogenolysis to gluconeogenesis, fat oxidation and ketogenesis [30,31,32]. Studies suggest that moderate- to high-intensity exercise for ≥60 min performed in the fasted state may be the most effective combination to induce this metabolic switch [30,31,32,104,105]. Moreover, implementing structured endurance exercise may be an effective strategy to avoid the reduction in low- to moderate-intensity activity energy expenditure observed with IF regimens [42,97,100]. Another undesirable effect of IF is the potential loss of fat-free mass, which might be even more worrisome than in continuous daily CR [42,63]. This undesirable effect is likely counteracted, at least in part, by resistance exercise.

The study of IF in humans is still in its infancy. To date, pioneer studies have shown that IF regimens slightly decrease body weight and fat mass and improve cardiometabolic risk factors compared to unrestricted eating, especially in metabolic unhealthy study participants [33,34,35,36,37,49,50]. According to the current evidence, IF regimens and continuous daily CR seem to be equally effective to enhance body composition and cardiometabolic health [34,40], which is partly explained by the similar adherence to both interventions [7,40,41]. However, one crossover study (5 weeks for each intervention) in our lab found that eucaloric (i.e., energy intake equals energy needs, and thus in absence of weight loss) early TRE improved appetite regulation, blood pressure, oxidative stress, insulin sensitivity, and β cell responsiveness in men with prediabetes [8]. These observations suggest that IF regimens may improve cardiometabolic health independently of weight loss. Little is known about the effects of IF regimens on ectopic fat accumulation and postprandial metabolism, two important determinants of metabolic health. The few studies conducted reported no differences between IF regimens and continuous daily CR except for the reduction in postprandial triglycerides, which seems to be enhanced in IF regimens [42,54]. Together, preliminary evidence from human trials suggests that the cardiometabolic health benefits of IF are mediated, at least in part, by the mentioned adaptations in energy metabolism. Nonetheless, properly powered, long-term well-controlled clinical research is still needed to unravel the underlying mechanisms of IF and its role on energy metabolism including energy expenditure and fat oxidation.

## Figures and Tables

**Table 1:** Chronic effects of Ramadan on energy expenditure and substrate oxidation.

Study	Population	Design and Intervention	Assessment	No Change	Increase	Decrease
[71]	Normal weight *n* = 16 (0 M/16 F)	4-week Observational (1) Ramadan	15 h period Metabolic chamber	RMR and nigh EE Day and evening protein ox.	Fat ox. from 2 p.m.–11 p.m.	RER and CHO ox. from 11 a.m.–11 p.m. EE from 11 a.m.–5 p.m.
[73]	(1) Healthy *n* = 7 (7 M/0 F) (2) TD2M *n* = 5 (3 M/2 F)	3-week Observational (1) Ramadan (2) Ramadan	Overnight fasting Metabolic cart	RMR and RER		
[72]	Healthy normal/overweight *n* = 29 (13 M/16 F) for RMR *n* = 10 (5 M/5 F) for TDEE	4-week Observational (1) Ramadan	Overnight fasting Metabolic cart (Quark, Cosmed) Doubly-labeled water during 14 days	RMR and adjusted RMR * TDEE		RER

No change indicates no significant difference to pre-intervention values; increase indicates significantly higher than pre-intervention values; and decrease indicates significantly lower than pre-intervention values. * RMR results adjusted for sex, age, weight, and the number of hours since suhoor. Metabolic cart brands and models are provided when they are available. Abbreviations: EE, energy expenditure; RER, respiratory exchange ratio; RMR, resting energy expenditure; TDEE, total daily energy expenditure; TD2M, type 2 diabetes mellitus.
